# Overexpression of the PP2A regulatory subunit Tap46 leads to enhanced plant growth through stimulation of the TOR signalling pathway

**DOI:** 10.1093/jxb/eru438

**Published:** 2014-11-15

**Authors:** Chang Sook Ahn, Hee-Kyung Ahn, Hyun-Sook Pai

**Affiliations:** Department of Systems Biology, Yonsei University, Seoul 120-749, Korea

**Keywords:** Kinematic analysis, phosphorylation of S6 kinase, PP2A catalytic subunit, rapamycin, real-time quantitative PCR, seed viability.

## Abstract

Tap46, a regulatory subunit of protein phosphatase 2A (PP2A), plays an essential role in plant growth and development through a functional link with the Target of Rapamycin (TOR) signalling pathway. Here, we have characterized the molecular mechanisms behind a gain-of-function phenotype of Tap46 and its relationship with TOR to gain further insights into Tap46 function in plants. Constitutive overexpression of Tap46 in *Arabidopsis* resulted in overall growth stimulation with enlarged organs, such as leaves and siliques. Kinematic analysis of leaf growth revealed that increased cell size was mainly responsible for the leaf enlargement. Tap46 overexpression also enhanced seed size and viability under accelerated ageing conditions. Enhanced plant growth was also observed in dexamethasone (DEX)-inducible Tap46 overexpression *Arabidopsis* lines, accompanied by increased cellular activities of nitrate-assimilating enzymes. DEX-induced Tap46 overexpression and *Tap46* RNAi resulted in increased and decreased phosphorylation of S6 kinase (S6K), respectively, which is a sensitive indicator of endogenous TOR activity, and Tap46 interacted with S6K *in planta* based on bimolecular fluorescence complementation and co-immunoprecipitation. Furthermore, inactivation of TOR by estradiol-inducible RNAi or rapamycin treatment decreased Tap46 protein levels, but increased PP2A catalytic subunit levels. Real-time quantitative PCR analysis revealed that Tap46 overexpression induced transcriptional modulation of genes involved in nitrogen metabolism, ribosome biogenesis, and lignin biosynthesis. These findings suggest that Tap46 modulates plant growth as a positive effector of the TOR signalling pathway and Tap46/PP2Ac protein abundance is regulated by TOR activity.

## Highlight

Our study of Tap46 overexpression suggests that Tap46 enhances plant growth as a positive effector of the TOR signalling pathway. Furthermore, the abundance of Tap46/PP2Ac protein is regulated by TOR activity.

## Introduction

Target of Rapamycin (TOR) is a Ser/Thr protein kinase that is evolutionarily conserved among all eukaryotes. The TOR signalling pathway integrates diverse signals, such as nutrients, energy, growth factors, and environmental conditions, to regulate cell growth and metabolism in yeast, plants, and mammals (Hay and Sonenberg, 2004; Wullschleger *et al.*, 2006; Bögre *et al.*, 2013; Xiong and Sheen, 2013). In yeast, TOR kinase becomes activated under favourable conditions to promote ribosome biogenesis, protein translation, transcription, and nutrient uptake, while rapamycin treatment or nutrient deficiency inactivates TOR, turning off the processes and activating autophagy, proteolysis, and stress responses instead (Loewith, 2010). In mammals, TOR signalling similarly regulates metabolic activity and cell growth in response to diverse external signals, and has been linked to amino acid sensing and the insulin signalling pathway (Zoncu *et al.*, 2011; Laplante and Sabatini, 2012). TOR kinase forms two distinct multiprotein complexes in yeast and mammals, named TOR complex 1 (TORC1) and TOR complex 2 (TORC2), which contain both conserved and distinct protein components. Rapamycin-sensitive TORC1 is mainly involved in the control of metabolism, translation, and autophagy, while rapamycin-insensitive TORC2 regulates cytoskeleton structure, apoptosis, and transcription (Wullschleger *et al.*, 2006; Zoncu *et al.*, 2011; Shimobayashi and Hall, 2014).

In contrast to yeast and mammalian systems, molecular studies on the plant TOR signalling pathway have been impeded by its reduced sensitivity to rapamycin (Soulard *et al.*, 2009). Moreover, disruption of plant TOR-pathway genes, such as *TOR*, *Tap46*, and *RAPTOR,* is lethal to the embryo (Menand *et al.*, 2002; Deprost *et al.*, 2005; Ahn *et al.*, 2011). Expression of FKP12, which mediates rapamycin binding to TOR, renders *Arabidopsis* plants rapamycin sensitive, providing an invaluable tool for dissecting the signalling pathway in higher plants (Sormani *et al.*, 2007; Ren *et al.*, 2012; Xiong and Sheen, 2012). Using this system, Xiong *et al.* (2013) reported that TOR senses and transduces shoot photosynthesis-derived glucose signals that control cell proliferation in the root meristem through transcriptional reprogramming of numerous genes involved in metabolism and biosynthesis for energy production and growth. Furthermore, TOR directly phosphorylates the E2Fa transcription factor, which can then activate transcription of S-phase genes, a novel function for TOR in direct transcriptional regulation of the cell cycle (Xiong *et al.*, 2013). The phytohormone auxin activates TOR kinase, promoting S6K1 phosphorylation at T449 and translation reinitiation of upstream open reading frame (uORF)-containing mRNAs via phosphorylation of eIF3h (eukaryotic initiation factor 3, subunit h) (Schepetilnikov *et al.*, 2013).

Tap42/α4, a regulatory subunit of protein phosphatase 2A, is a downstream effector of TOR kinase in yeast and mammals. Tap42/α4 directly associates with the catalytic subunits of PP2A and the PP2A-related phosphatases PP4 and PP6 to form a heterodimer (Chen *et al.*, 1998; Düvel and Broach, 2004). Yeast Tap42 is phosphorylated by TOR and acts downstream of TOR in many TOR-mediated responses (Jacinto *et al.*, 2001; Dűvel and Broach, 2004). Loss of function of Tap42 mimics the effects of rapamycin treatment (Yan *et al.*, 2006). α4, the mammalian homologue of Tap42, regulates dephosphorylation of the transcription factors c-jun and p53. Deletion of α4 rapidly initiates apoptosis in proliferating, as well as differentiated, cells in mice (Kong *et al.*, 2004).

Tap46, a plant homologue of Tap42/α4, plays an essential role in plant cell growth and survival as a component of the TOR signalling pathway (Ahn *et al.*, 2011). Like Tap42/α4, Tap46 interacts with catalytic subunits of PP2A, PP4, and PP6, and recombinant Tap46 is phosphorylated by immuno-precipitated TOR kinase *in vitro*. Tap46-deficient plants exhibit the signature phenotypes of TOR inactivation, including repression of global translation and activation of nitrogen mobilization and autophagy. Taken together, these results suggest that Tap46 may function as a downstream effector of TOR signalling in the regulation of plant survival, growth, and development (Ahn *et al.*, 2011). In this study, we further investigated Tap46 functions in plants by characterizing its gain-of-function phenotype and its relationship with the TOR pathway. Tap46 overexpression in *Arabidopsis* stimulated overall plant growth resulting in enlarged plants with larger leaves and seeds, and increased nitrogen-assimilating activities. Increased phosphorylation of S6 kinase (S6K) was detected in Tap46 overexpression plants, suggesting activation of the TOR signalling pathway. Furthermore, TOR activity oppositely modulated protein abundance of Tap46 and the PP2A catalytic subunit (PP2Ac), suggesting their antagonistic relationship in the TOR signalling pathway.

## Materials and methods

### Plant material and growth conditions

*Arabidopsis thaliana* wild type (WT; ecotype Col-0) and the *Tap46* overexpression transgenic plants in the Col-0 background were grown in a growth room at 23°C and 60 μmol m^–2^ s^–1^ light intensity under a 16h light/8h dark cycle. For rapamycin treatment, seeds were germinated and grown in six-well plates containing 1ml of liquid medium (0.5 X MS and 0.5% sucrose, pH 5.7) with 1 or 10 μM rapamycin.

### Kinematic analysis of leaf growth

Kinematic analysis was performed as described previously (De Veylder *et al.*, 2001; Gonzalez *et al.*, 2010). For the leaf area analysis, first leaves were harvested from seven to eight plants grown in soil at 4 to 25 days after cotyledon emergence. The leaves were cleared with clearing solution [3:1 (v/v) ethanol:acetic acid] and mounted on microscope slides for photography, and the leaf area was determined with ImageJ software (http://rsb.info.nih.gov/ij/). Abaxial epidermal cells (40–100 cells) were drawn for four to five leaves with a light microscope (Olympus) fitted with a drawing tube and a differential interference contrast objective. Photographs of leaves and drawings were used to measure the leaf area and the average cell area with the ImageJ software. Epidermal cell numbers per leaf were calculated using the leaf area and average cell area.

### Immunoblotting

Protein extracts (30 µg) were subjected to SDS-PAGE and immunoblotting as described previously (Ahn *et al.*, 2011; Lee *et al.*, 2013). Anti-Tap46 antibodies were generated in rabbits against an oligopeptide, NQPLIFGPASIVGGP, that corresponds to amino acid residues of *Arabidopsis* Tap46 at positions 289 to 303 using the antibody production services of Cosmogenetech (http://www.cosmogenetech.com). Immunoblotting was performed using mouse monoclonal antibodies against the haemagglutinin (HA) tag (1:10 000 dilution; Applied Biological Materials), the Myc tag (1:10 000 dilution; Applied Biological Materials), and the Flag tag (1:10 000; Sigma), or using rabbit polyclonal antibodies against α-tubulin (1:1000 dilution; Sigma), the PP2A catalytic subunit (1:1000; Cell Signalling), and Tap46 (1:5000; Cosmogenetech). The membranes were then treated with horseradish peroxidase-conjugated goat anti-mouse IgG antibodies (1:10 000; Invitrogen) or goat anti-rabbit antibodies (1:10,000; Invitrogen), respectively. Signals were detected on X-ray film (Kodak) using an ECL chemiluminescence kit (ELPIS-Biotech, Inc.).

### Detection of S6K phosphorylation

Protoplast transient expression assays were carried out as described previously (Xiong and Sheen, 2012). Flag-tagged WT or mutant S6K1 (T449A) was transiently expressed in mesophyll protoplasts isolated from the transgenic *Arabidopsis* seedlings grown in (–)DEX or (+)DEX medium (DEX: dexamethasone). Protoplasts were harvested by centrifugation and the protein extract was subjected to immunoblotting with anti-phospho-p70 S6K (pThr-389) polyclonal antibody (1:1000; Cell Signalling) to detect phosphorylation of the Thr-449 residue in *Arabidopsis* S6K1 and Thr-455 residue in *Arabidopsis* S6K2 as described (Xiong and Sheen, 2012).

### Other techniques

The following techniques are described in Supplementary Methods S1: generation of DEX-inducible Tap46 overexpression lines in *Arabidopsis*; *Agrobacterium*-mediated transient expression; real-time quantitative PCR; bimolecular fluorescence complementation (BiFC); co-immunoprecipitation; measurement of enzyme activities; accelerated-ageing treatment and tetrazolium staining of seeds; and statistical analyses.

## Results

### Overexpression of Tap46 leads to enhanced plant growth

**Fig. 1.**
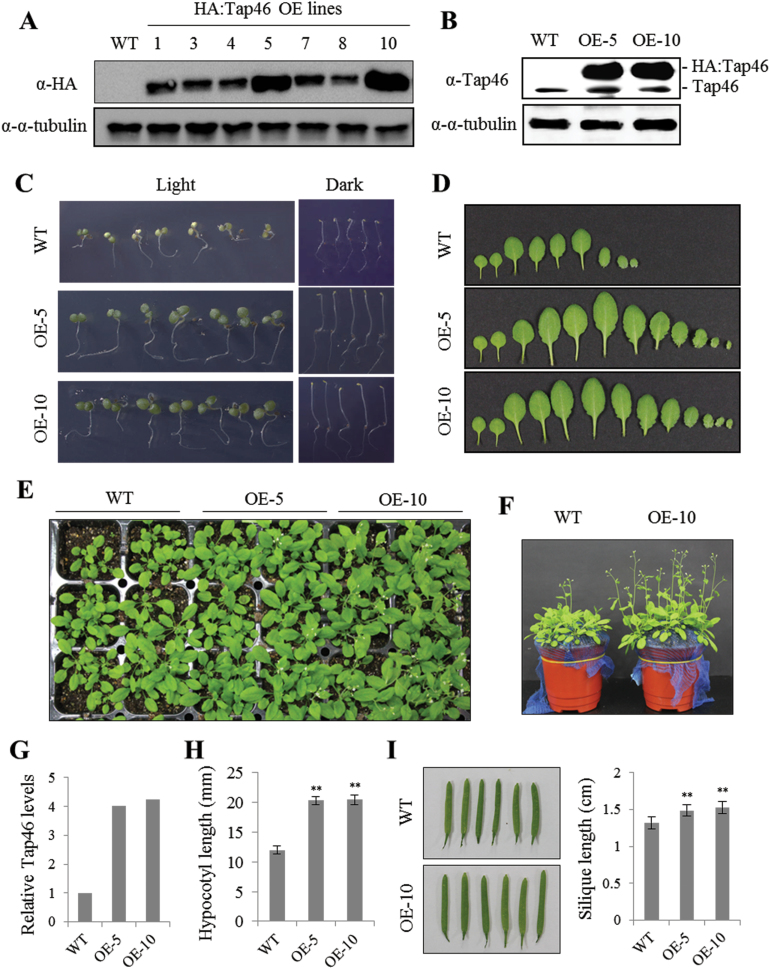
Enhanced growth of *Arabidopsis* Tap46 constitutive overexpression (OE) lines. (A) Immunoblotting with anti-HA antibodies to measure expression of HA:Tap46 fusion protein in WT (Col-0) and seven independent Tap46 OE lines. Thirty micrograms of total protein isolated from seedlings were subjected to immunoblotting. Anti-α-tubulin antibody was used as a loading control. (B) Immunoblotting with anti-Tap46 antibodies of protein extracts from WT and *Tap46* OE (OE-5 and OE-10) seedlings. (C) Seedling growth of WT, OE-5, and OE-10 lines on MS medium under light and dark conditions 4 days after sowing. (D) Representative leaf series of WT, OE-5, and OE-10 plants grown for 3 weeks in soil. (E) WT, OE-5, and OE-10 plants grown for 3 weeks in soil. (F) WT and OE-10 plants grown for 4 weeks in soil. (G) Relative expression levels of Tap46 protein in WT, OE-5, and OE-10 seedlings based on quantification of the immunoblot band intensities in (B). Levels of endogenous and HA-tagged Tap46 were combined for the OE lines. (H) Average hypocotyl length of WT and Tap46 OE seedlings grown under dark conditions 4 days after sowing (*n* = 30). Asterisks denote the statistical significance of the differences between WT and Tap46 OE samples: *, *P* ≤ 0.05; **, *P* ≤ 0.01. (I) Average silique length of WT and Tap46 OE lines (*n* = 30). This figure is available in colour at *JXB* online.

To investigate the role of Tap46 in plant growth, we generated transgenic *Arabidopsis* lines (Col-0 ecotype) that constitutively overexpress Tap46 fused to an HA tag under the control of a cauliflower mosaic virus 35S promoter. The presence of the *HA:Tap46* transgene was confirmed by genomic PCR in >30 independent T1 transgenic lines. Ten T2 lines containing a single copy transgene were selected. T3 homozygous plants of most of these lines exhibited enhanced growth compared with the WT plants. Immunoblotting with anti-HA antibody using cell extracts from the T3 lines confirmed the expression of HA:Tap46 proteins (Fig. 1A). Two overexpression lines, OE-5 and OE-10, were selected for further study based on strong phenotypes and high HA:Tap46 expression indicated by immunoblotting with anti-HA and anti-Tap46 antibodies (Fig. 1A, B). Quantification of the immunoblot bands in Fig. 1B showed that total Tap46 protein levels in OE-5 and OE-10 leaves were ~4-fold higher than the levels in WT plants (Fig. 1G). The Tap46 overexpression seedlings exhibited enhanced cotyledon and root growth on Murashige and Skoog (MS) medium under light conditions (Fig. 1C). Dark-grown Tap46 overexpression seedlings also exhibited longer hypocotyls and roots (Fig. 1C): the hypocotyls of dark-grown OE-5 and OE-10 seedlings were ~1.8-fold longer than those of WT seedlings (Fig. 1H). Leaf series of plants grown in soil for 3 weeks showed an increase in size and number in OE-5 and OE-10 plants, suggesting enhanced plant growth (Fig. 1D). OE-5 and OE-10 plants grew bigger and faster, and reached maturity earlier, than WT plants (Fig. 1E, F). At maturity, the overexpression plants had enlarged leaves and siliques: for example, siliques of the overexpression plants were ~15–20% longer than those of WT plants (Fig. 1F, I). Thus, Tap46 overexpression affected the mechanisms that regulate plant growth rates and final organ size.

### Kinematic analysis of leaf growth

**Fig. 2.**
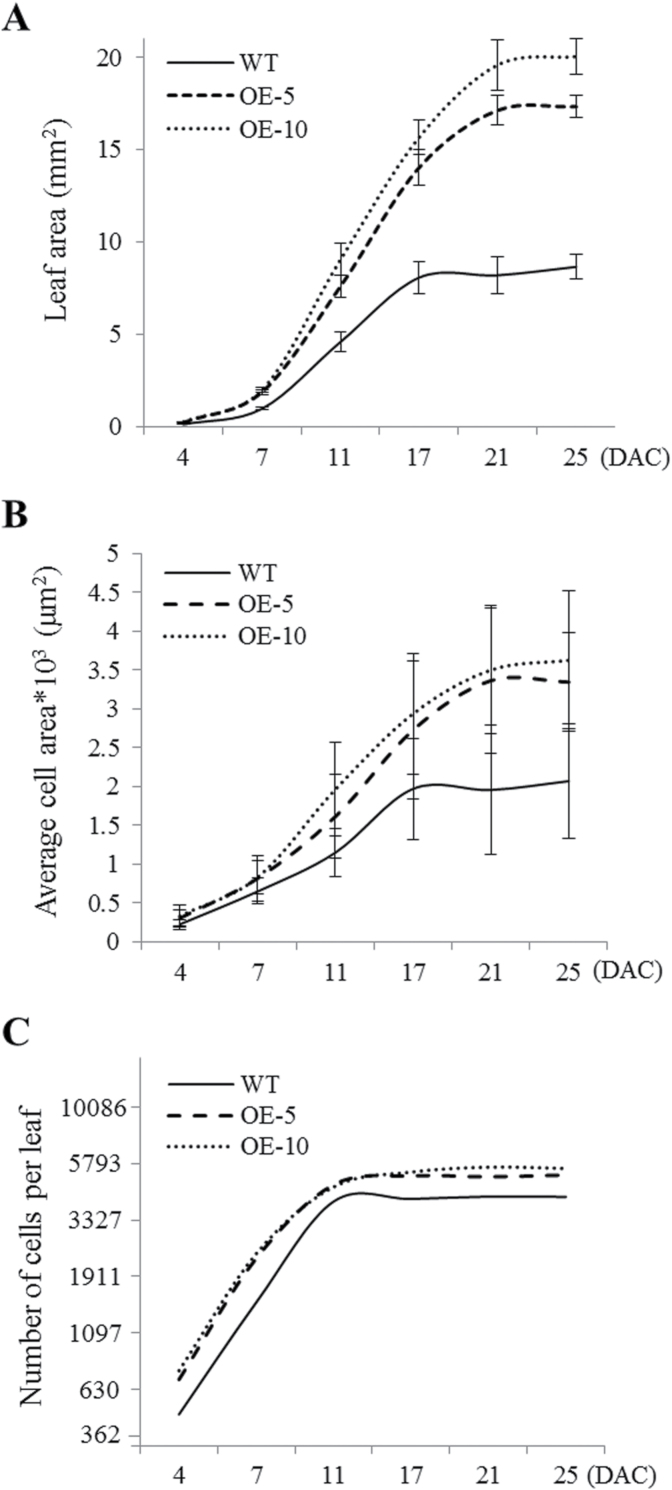
Kinematic analysis of leaf growth. Seeds were imbibed at 4°C for 24 hours before sowing. First leaves were collected from WT, OE-5, and OE-10 plants at 4, 7, 11, 17, 21, and 25 DAC. (A) Average areas of first leaves. (B) Average areas of epidermal cells on the abaxial side of first leaves. (C) Calculated numbers of epidermal cells per leaf.

Final organ size is determined by two parameters: cell division and cell expansion (Horiguchi *et al.*, 2006; Anastasiou and Lenhard 2007). To gain insight into the effect of Tap46 overexpression on the two parameters during leaf development, kinematic analysis was performed on the first leaves of OE-5 and OE-10 transgenic plants in comparison with those of WT plants (Fig. 2; Supplementary Table 1; Supplementary Figure S1). First leaves of seven to eight soil-grown plants were harvested at 4, 7, 11, 17, 21, and 25 days after cotyledon emergence (DAC) to assess leaf size and abaxial epidermal cell size, and to calculate the epidermal cell number using the two values. The leaf area of WT and Tap46 overexpression lines progressively increased to reach a plateau at 17 and 21 DAC, respectively. Increased leaf size of Tap46 overexpression plants was visible as early as 4 DAC, and the final leaf size was 2.0 to 2.31-fold larger than that of the WT at 25 DAC (Fig. 2A; Supplementary Table 1). Tap46 overexpression leaves had larger epidermal cells at 4 DAC compared with WT leaves, and the final cell size of Tap46 overexpression leaves at 25 DAC was 1.62- to 1.75-fold larger than that of WT leaves (Fig. 2B; Supplementary Table 1). Furthermore, the leaf epidermal cells of Tap46 overexpression lines reached their final sizes at 21 DAC, while those of the WT reached a plateau at 17 DAC, suggesting prolonged cell expansion in Tap46 overexpression leaves. When the first leaves of WT and Tap46 overexpression lines were observed at 2 DAC, there was no difference in epidermal cell sizes, suggesting that cell division occurs in a similar size in WT and Tap46 overexpression lines (Supplementary Figure S2). The epidermal cell number per leaf increased rapidly during the early stages and then remained nearly constant from 11 DAC onward for both WT and Tap46 overexpression plants. The estimated cell number of OE-5 and OE-10 leaves was 1.23-fold and 1.32-fold higher than that of WT plants at 25 DAC (Fig. 2C; Supplementary Table 1). Taken together, these results suggest that the leaf size enlargement of Tap46 overexpression plants is mainly caused by cell expansion, occurring more vigorously for an extended period of time, although increased cell division also contributes to the increased leaf size.

### Seed enlargement in Tap46 overexpression plants

**Fig. 3.**
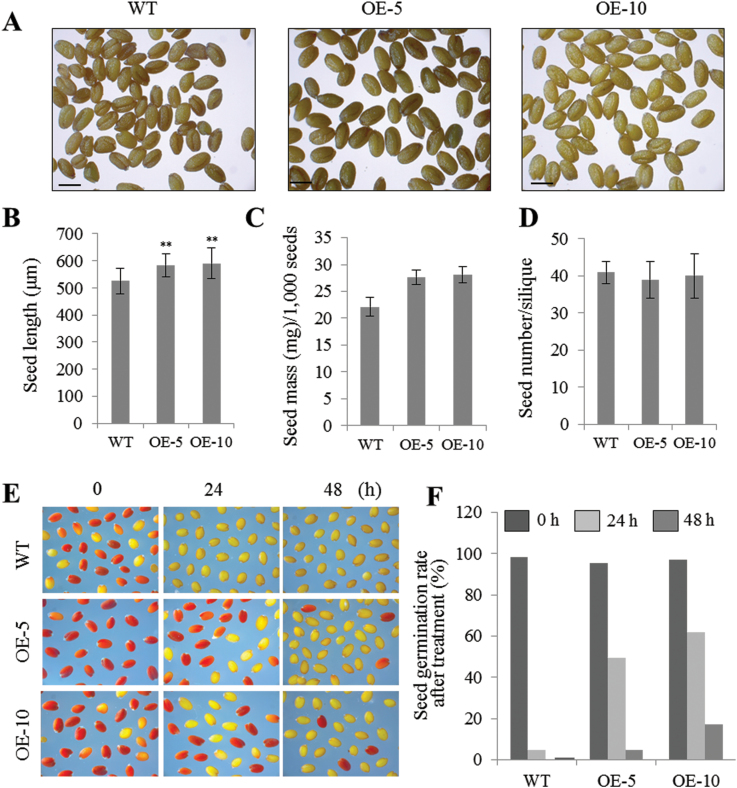
Analysis of seed enlargement in Tap46 overexpression lines. (A) Mature seeds of WT, OE-5, and OE-10 plants. Scale bars, 500 µm. (B) Average seed length (*n* = 500). (C) Average seed mass per 1000 seeds (*n* = 3). (D) Average seed number per silique (*n* = 30). (E) Tetrazolium staining to determine seed viability. WT, OE-5, and OE-10 seeds were incubated under accelerated ageing conditions for 0–48 hours. Viable seeds are stained red (and appear here as the darker seeds). (F) Germination rate (%) of WT, OE-5, and OE-10 seeds after accelerated ageing treatment for 0–48 hours (*n* = 100). This figure is available in colour at *JXB* online.

To investigate the effect of Tap46 overexpression on seed development, we determined the average length and mass of mature dry seeds from WT, OE-5, and OE-10 plants that had been grown in soil under the same conditions. OE-5 and OE-10 seeds were longer and heavier than WT seeds (Fig. 3A–C), although the seed number per silique was slightly reduced in Tap46 overexpression plants (Fig. 3D). To test seed quality, seed viability was monitored under accelerated-aging conditions (Fig. 3E, F). Seeds were incubated under high temperature (43°C) and high humidity (100% relative humidity) for 0–72 hours. Tetrazolium uptake assays were performed to determine seed viability. Upon incubation with the tetrazolium solution, viable seeds are stained red, while seeds with low viability remain white (Oge *et al.*, 2008; Seo *et al.*, 2011). OE-5 and OE-10 seeds survived and germinated at a higher frequency after 24 or 48 hours of treatment, compared with WT seeds (Fig. 3E, F). None of the WT or Tap46 overexpression seeds survived after 72 hours of treatment. Thus, Tap46 overexpression seeds appeared to be more resistant to environmental stresses that reduce seed viability.

### DEX-inducible overexpression of Tap46 results in enhanced plant growth

**Fig. 4.**
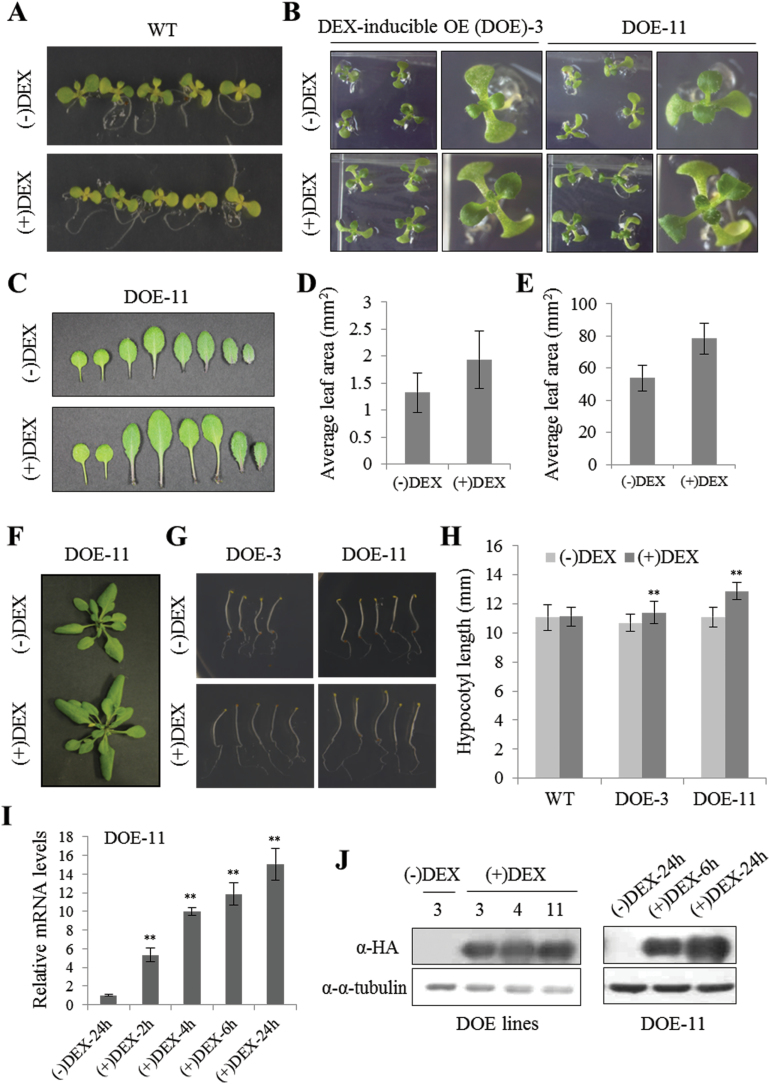
Enhanced growth of *Arabidopsis* DEX-inducible Tap46 overexpresion (DOE) lines. (A) WT seedling growth on MS medium containing ethanol [(–)DEX] or 10 μM DEX [(+)DEX] at 6 DAS. (B) Seedling growth of DOE-3 and DOE-11 lines on (–)DEX and (+)DEX medium 6 DAS. (C) Representative leaf series of soil-grown DOE-11 plants that were sprayed with ethanol or 30 µM DEX for 16 DAG. (D) Average area of the first leaves of soil-grown DOE-11 plants that were sprayed with ethanol or DEX for 4 days after germination. (E) Average area of the largest rosette leaves of soil-grown DOE-11 plants that were sprayed with ethanol or DEX for 16 days after germination. (F) Soil-grown DOE-11 seedlings sprayed with ethanol or DEX for three weeks. (G) Seedling growth 4 DAS on (–)DEX or (+)DEX medium under dark conditions. (H) Average hypocotyl length of dark-grown seedlings on (–)DEX and (+)DEX medium 4 DAS (*n* = 30). (I) Real-time quantitative RT-PCR analysis of *Tap46* transcript levels. Transcript levels in (+)DEX samples are expressed relative to those in (–)DEX-24 hour samples. Values represent means ±SD of three replicates per experiment. (J) Immunoblotting to measure accumulation of HA:Tap46 fusion protein in DOE lines. Thirty micrograms of total protein isolated from different DOE lines (nos. 3, 4, and 11) with or without DEX treatment for 24 hours were subjected to immunoblotting with anti-HA antibodies (left). HA:Tap46 protein accumulation was detected by immunoblotting in DOE-11 seedlings after 6 and 24 hours of DEX treatment (right). Anti-α-tubulin antibody was used as a loading control. This figure is available in colour at *JXB* online.

To confirm that Tap46 overexpression leads to enhanced seedling growth, we generated dexamethasone (DEX)-inducible *Tap46* overexpression (DOE) lines in *Arabidopsis* that carry the *HA:Tap46* transgene under the control of a DEX-inducible transcription system (Aoyama and Chua, 1997) (Fig. 4). WT and transgenic seedlings were grown in MS medium containing ethanol [(–)DEX] or 10 µM DEX [(+)DEX]. Upon DEX treatment, seedlings of two independent DOE lines (no. 3 and no. 11) showed enhanced shoot growth compared with (–)DEX DOE or WT seedlings with or without DEX treatment (Fig. 4A, B). Leaf series of soil-grown DOE-11 plants 16 days after germination (DAG) demonstrated that DEX spraying resulted in an increase in leaf size compared with (–)DEX plants (Fig. 4C). Quantification revealed that DEX spraying increased the average area of the first leaves (4 DAG) and the largest leaves (16 DAG) of DOE-11 plants, suggesting that Tap46 overexpression stimulates plant growth (Fig. 4D, E). Prolonged DEX spraying of soil-grown DOE lines resulted in enlarged plants (Fig. 4F). DEX-treated DOE seedlings also grew slightly faster than ethanol-treated seedlings under dark conditions, resulting in longer hypocotyls at 4 days after sowing (DAS; Fig. 4G, H). A time-dependent effect of DEX treatment on *Tap46* mRNA levels was determined by real-time quantitative RT-PCR using *Tap46*-specific primers described in Supplementary Table S2 (Fig. 4I). DOE-11 seedlings were grown on MS medium for 10 days, transferred to new MS medium containing ethanol [(–)DEX] or 10 μM DEX [(+)DEX], and incubated for 2–24 hours. DOE-11 seedlings exhibited a progressive increase in *Tap46* transcript levels upon DEX treatment compared with the (–)DEX 24-h sample (Fig. 4I). Immunoblotting with anti-HA antibodies detected ectopic expression of the HA:Tap46 fusion protein in the transgenic DOE-3, DOE-4, and DOE-11 lines upon DEX treatment for 24 hours (Fig. 4J, left). A time-dependent accumulation of HA:Tap46 protein in DOE-11 lines upon DEX treatment is shown in Fig. 4J (right). These results further support the hypothesis that Tap46 overexpression stimulates plant growth.

### Activities of nitrogen metabolic enzymes

In aerobic soils, the major form of inorganic nitrogen is nitrate (Xu *et al.*, 2012). A large portion of nitrate absorbed by the roots is transported to the shoot and reduced to nitrite by nitrate reductase (NR) and then to ammonium by nitrite reductase (NiR). Ammonium derived from nitrate or directly absorbed via ammonium transporters is further assimilated into amino acids by the glutamine synthetase (GS)/glutamine 2-oxoglutarate aminotransferase (GOGAT) cycle (Xu *et al.*, 2012). Induction of *TOR* RNAi in *Arabidopsis* results in 2- to 3-fold higher levels of GS and glutamate dehydrogenase activities that are usually associated with leaf senescence and nutrient recycling (Deprost *et al.*, 2007). Activation of *Tap46* RNAi resulted in reduced NR activity (Ahn *et al.*, 2011).

**Fig. 5.**
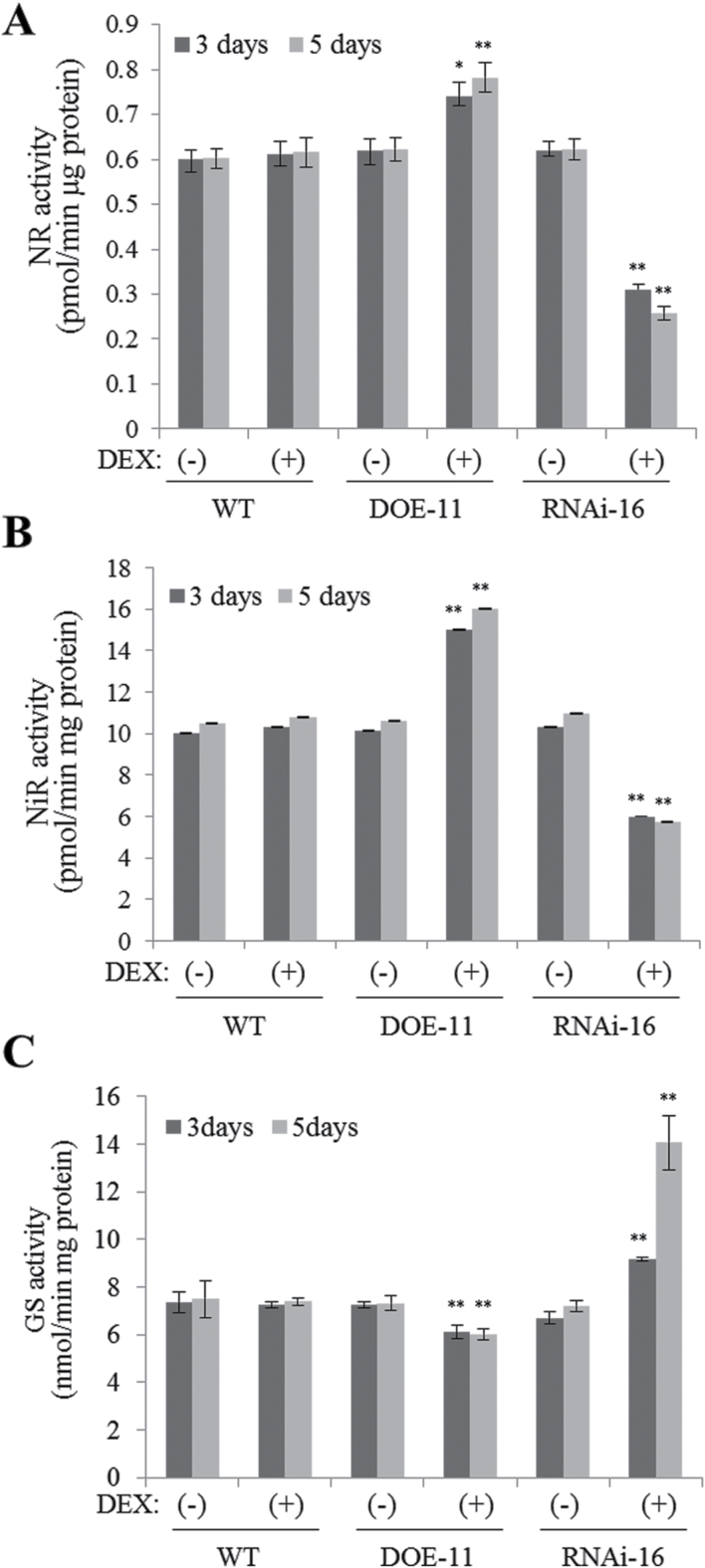
Modulation of metabolic enzyme activities in DEX-inducible *Tap46* overexpression and *Tap46* RNAi (RNAi-16) plants. Enzyme activities were measured using protein fractions prepared from WT, DOE-11, and RNAi-16 seedlings that were grown in soil for 2 weeks and sprayed with ethanol [(–)DEX] or 30 μM DEX [(+)DEX] for 3 or 5 days. Data points represent mean ± SD of three experiments. Asterisks denote statistical significance of the differences between (–)DEX and (+)DEX samples on each day: *, *P* ≤ 0.05; **, *P* ≤ 0.01. (A) NR activities. (B) NiR activities. (C) GS activities.

We examined the activities of several key nitrogen-assimilating enzymes in the DEX-inducible *Tap46* overexpression (DOE-11) and DEX-inducible *Tap46* RNAi (RNAi-16) lines (Ahn *et al.*, 2011) (Fig. 5). Seedlings were grown for 2 weeks in MS medium and transferred to new MS medium containing ethanol [(–)DEX] or 10 µM DEX [(+)DEX] for further growth for 3 or 5 days. Increased total NR activities were detected in DOE-11 seedlings upon DEX treatment, while NR activities of DEX-treated RNAi-16 seedlings were lower than those of (–)DEX samples (Fig. 5A). There were no differences in the activity between WT (–)DEX and (+)DEX samples. To assess how much of the NR enzyme is in the non-phosphorylated active form, NR activation state was determined by comparing NR activity measured in the presence of Mg^2+^ with the activity measured in the presence of EDTA (total activity) (Supplementary Table S3; Lillo *et al.*, 2003). NR activation state was only slightly increased in DOE-11 seedlings after DEX treatment for 3 and 5 days, while DEX treatment of WT seedlings did not change the activation state. These results suggest that the moderate increase (~25%) in total NR activity observed in DEX-treated DOE-11 seedlings is mainly caused by increased NR protein levels. NiR activities increased and decreased in DOE-11 and RNAi-16 lines, respectively, upon DEX treatment (Fig. 5B). These results suggest that the increased NR and NiR activities contributed to enhanced growth of the transgenic plants. GS activity, however, was slightly reduced in DOE-11 seedlings after 3 and 5 days of DEX treatment, while the activity significantly increased in DEX-treated RNAi-16 seedlings (Fig. 5C). GS functions as the major assimilatory enzyme for ammonium, but it also plays a role in re-assimilation of recycled nitrogen released from protein degradation during senescence (Pageau *et al.*, 2006; Xu *et al.*, 2012). Increased GS activity in the RNAi-16 lines is consistent with the early senescence phenotype and increased glutamine accumulation observed in the plants (Ahn *et al.*, 2011).

### Stimulated TOR signalling pathway in Tap46 DOE plants

**Fig. 6.**
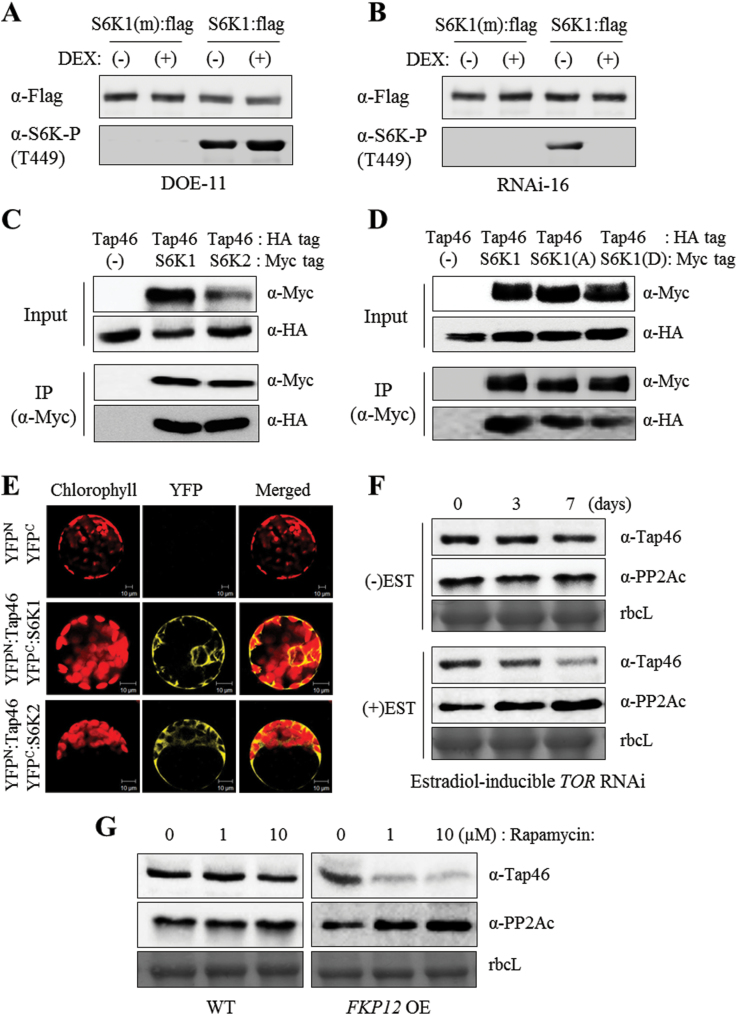
Determination of the relationship between Tap46 and the TOR signalling pathway. (A) Phosphorylation of S6K1 and its mutant in DOE-11 seedlings upon DEX treatment. Flag-tagged WT S6K1 or mutant S6K1 [S6K1(m); T449A, non-phosphorylated form] were transiently expressed in protoplasts. Western blot analyses were performed with anti-Flag antibodies to detect total S6K1 protein and with anti-S6K1-P(T449) antibodies to detect S6K1 phosphorylated at T449. (B) Phosphorylation of S6K1 and its mutant in DEX-inducible *Tap46* RNAi-16 seedlings upon DEX treatment. (C) Co-immunoprecipitation. Protein extracts were prepared from *N. benthamiana* leaves that expressed HA:Tap46 with S6K1:Myc or S6K2:Myc fusion proteins. After immunoprecipitation (IP) with anti-Myc antibodies, co-immunoprecipitated HA:Tap46 was detected by immunoblotting with anti-HA antibodies. To check IP efficiency, the precipitated fractions were also immunoblotted with anti-Myc antibodies (input). (D) Co-immunoprecipitation of HA:Tap46 with S6K1:Myc, S6K1(A):Myc, or S6K1(D):Myc fusion proteins. S6K1(T449A) is a non-phosphorylated form and S6K1(T449D) is a phospho-mimetic form. (E) Visualization of interactions of Tap46 with S6K1 and S6K2 using bimolecular fluorescence complementation (BiFC). YFP^N^:Tap46 was expressed together with YFP^C^:S6K1 or YFP^C^:S6K2 fusion proteins in *N. benthamiana* leaves by agroinfiltration for confocal laser scanning microscopy. (F) Immunoblot analysis to detect cellular Tap46 and PP2Ac protein levels in *TOR* RNAi lines. Thirty micrograms of total protein isolated from *Arabidopsis* estradiol-inducible *TOR* RNAi seedlings after 3 and 7 days of ethanol (–EST) or 10 µM estradiol (+EST) treatment were subjected to immunoblotting with anti-Tap46 and anti-PP2Ac antibodies. Coomassie-stained Rubisco large subunit (rbcL) is shown as a loading control. (G) Immunoblot analysis to detect cellular Tap46 and PP2Ac protein levels in WT and *Arabidopsis FKP12* overexpression lines upon rapamycin treatment. Seedlings were treated with 1 or 10 µM rapamycin for 24 hours prior to immunoblotting. This figure is available in colour at *JXB* online.

Mammalian TOR (mTOR) regulates cap-dependent translation machinery through phosphorylation of its downstream substrate S6K and 4E-BP in response to nutrients, energy, and environmental stresses (Ma and Blenis, 2009). Inactivation of mTOR kinase by rapamycin leads to dephosphorylation of S6K, and PP2A was reported to interact with and dephosphorylate S6K in mammalian cells (Peterson *et al.*, 1999; Westphal *et al.*, 1999). In *Arabidopsis*, TOR activation triggers phosphorylation of S6K at specific sites (S6K1 at T449 and S6K2 at T455), suggesting that S6K is a true substrate of TOR kinase in plants (Schepetilnikov *et al.*, 2011; Xiong and Sheen, 2012). Since Tap46, a regulatory subunit of PP2A, functions as a downstream effector of plant TOR (Ahn *et al.*, 2011), we investigated whether Tap46 overexpression or *Tap46* RNAi affects S6K phosphorylation (Fig. 6A, B). DEX-inducible Tap46 overexpression (DOE-11) and *Tap46* RNAi (RNAi-16) lines were grown for 2 weeks in MS medium and transferred to new MS medium containing ethanol [(–)DEX] or 10 µM DEX [(+)DEX] for 3 days. Flag-tagged WT S6K1 or mutant S6K1 [S6K1(m); T449A] were transiently expressed in protoplasts isolated from DOE-11 and RNAi-16 seedlings as previously described (Xiong and Sheen, 2012). Immunoblotting of the protein extracts with anti-Flag and anti-phospho-S6K antibodies, followed by quantification of the immunoblot band intensities using ImageJ (http://imagej.nih.gov/ij/), demonstrated that the levels of phosphorylated S6K1 at T449 increased and decreased in the DOE-11 and RNAi-16 plants, respectively, as compared with total S6K1 protein levels (Fig. 6A, B; Supplementary Figure S3A). The T449A mutation in S6K1 completely abrogated the phosphorylation (Figure 6A, B). Therefore, Tap46 expression positively correlated with S6K phosphorylation, supporting its function as a positive effector of the TOR signalling pathway.

To further examine the relationship between Tap46 and S6K, we examined the interaction of Tap46 with S6K using co-immunoprecipitation and BiFC (Fig. 6C–E). For co-immunoprecipitation, HA-fused Tap46 (HA:Tap46) was co-expressed with Myc-fused S6K1 (S6K1:Myc) or S6K2 (S6K2:Myc) in *Nicotiana benthamiana* leaves by agroinfiltration (Fig. 6C). Protein expression was detected by immunoblotting with anti-HA and anti-Myc antibodies (Input). The S6K1:Myc and S6K2:Myc proteins were immunoprecipitated from leaf extracts using anti-Myc antibodies. Following SDS-PAGE, western blotting was performed with anti-Myc antibodies to detect the immunoprecipitated S6K proteins and then with anti-HA antibodies to detect HA:Tap46 as a co-immunoprecipitant. As shown in Fig. 6C, HA:Tap46 was co-immunoprecipitated at similar levels by S6K1:Myc and S6K2:Myc, suggesting that Tap46 can interact with both S6K1 and S6K2. As a control, when expressed alone, HA:Tap46 was not immunoprecipitated by anti-Myc antibodies (Fig. 6C). To determine the effect of S6K1 phosphorylation on its association with Tap46, a similar co-immunoprecipitation assay was performed (Fig. 6D). HA:Tap46 co-expressed with Myc-fused WT S6K1, S6K1(T449A; non-phosphorylated form), or S6K1(T449D; phospho-mimetic form). S6K1:Myc protein and its mutants were immunoprecipitated from leaf extracts by anti-Myc antibodies. Immunoprecipitants were analysed by western blotting with anti-Myc and anti-HA antibodies. HA:Tap46 was co-immunoprecipitated at similar levels regardless of the S6K1 form, indicating that S6K1 may interact with Tap46 using a surface that excludes the T449 site (Fig. 6D).

We next performed BiFC assays to determine the interaction of Tap46 with S6K1 and S6K2 (Fig. 6E; Supplementary Figure S4). For BiFC, the pSPYNE-Tap46 construct was agroinfiltrated together with the pSPYCE-S6K1 or pSPYCE-S6K2 constructs into *N. benthamiana* leaves to co-express YFP^N^:Tap46 and YFP^C^:S6K1/2 fusion proteins. After 48 hours, mesophyll protoplasts prepared from the infiltrated leaves or epidermal cells of the infiltrated leaves were observed by confocal laser scanning microscopy. Co-expression of YFP^N^:Tap46 and YFP^C^:S6K1, and of YFP^N^:Tap46 and YFP^C^:S6K2, both resulted in strong cytosolic YFP fluorescence (Fig. 6E; Supplementary Figure S4). As a control, no fluorescence was detected in the leaf protoplasts co-expressing YFP^N^ and YFP^C^ (Fig. 6E), and in the epidermal cells co-expressing YFP^N^ and YFP^C^, YFP^N^:Tap46 and YFP^C^, YFP^N^ and YFP^C^:S6K1, or YFP^N^ and YFP^C^:S6K2 (Supplementary Figure S4). Previously, GFP:S6K2 fusion protein that was transiently expressed in tobacco BY-2 cells by particle bombardment was mainly localized to the nucleus, but a minor portion was present in the cytosol (Mahfouz *et al.*, 2006). We observed that GFP:S6K2 was localized to both the nucleus and the cytosol in *N. benthamiana* leaves (Supplementary Figure S5). These analyses suggest that Tap46 interacts with S6K1 and S6K2 predominantly in the cytosol.

We investigated how the protein levels of Tap46 and its partner, PP2Ac, are modulated upon TOR inactivation. Estradiol-inducible *Arabidopsis TOR* RNAi effectively downregulated TOR protein accumulation, leading to inhibition of seedling growth and phosphorylation of S6K1 and S6K2 (Xiong and Sheen, 2012). Furthermore, transgenic *Arabidopsis* plants overexpressing *Arabidopsis* FKP12 exhibited significantly enhanced sensitivity to rapamycin, a drug that inactivates TOR (Xiong and Sheen, 2012). These strategies were employed to modulate TOR activity. Estradiol-inducible *TOR* RNAi lines were grown for 10 days in MS medium and then transferred to new MS medium containing ethanol (–EST) or 10 µM estradiol (+EST) for further growth for 3 and 7 days. Immunoblotting was performed with the protein extracts using anti-Tap46 and anti-PP2Ac antibodies, and the immunoblot band intensities were measured using ImageJ. Protein levels of Tap46 and PP2Ac decreased and increased, respectively, in the *TOR* RNAi seedlings following estradiol treatment, whereas the control Rubisco large subunit (rbcL) levels remained constant (Fig. 6F; Supplementary Figure S3B). Thus, protein accumulation of Tap46 and PP2Ac are positively and negatively regulated, respectively, by TOR activity. Treatment of the *FKP12* overexpression seedlings with 1 and 10 µM rapamycin also resulted in a progressive decrease and increase of Tap46 and PP2Ac protein levels, respectively (Fig. 6G; Supplementary Figure S3C). These results provide evidence that Tap46 and PP2A function as positive and negative effectors of TOR signalling, respectively, in plants.

### Real-time quantitative RT-PCR analysis of gene expression

Transcriptome profiling of WT and *tor* mutant seedlings after a 2-hour glucose treatment at the photoautotrophic transition point identified numerous target genes of the glucose-TOR pathway in *Arabidopsis* (Xiong *et al.*, 2013). The upregulated genes included those involved in root meristem activation, the cell cycle, DNA synthesis, ribosome biogenesis, protein translation, anabolic processes, cell wall biosynthesis, protein folding, and nitrate and lipid transport. The repressed genes encoded metabolic enzymes in catabolic pathways such as β-oxidation and the glyoxylate cycle, and proteins involved in protein/amino acid/lipid degradation or autophagy (Xiong *et al.*, 2013). Comparative transcriptome analysis of rapamycin-treated WT and BP12-expressing *Arabidopsis* lines using RNA-seq also revealed a large number of differentially expressed genes (Ren *et al.*, 2012). In rapamycin-treated BP12 lines, the downregulated genes were associated with anabolic pathways, such as cell wall biosynthesis, photosynthesis, and inorganic nutrient transport, whereas upregulated genes were related to catabolic pathways and stress pathways (Ren *et al.*, 2012). In addition, *TOR* silencing by estradiol-inducible artificial microRNA resulted in transcript changes of the genes involved in the cell cycle, cell wall modification, stress response, senescence, and primary and secondary metabolism, accompanied by major changes in metabolite profiles (Caldana *et al.*, 2013).

**Fig. 7.**
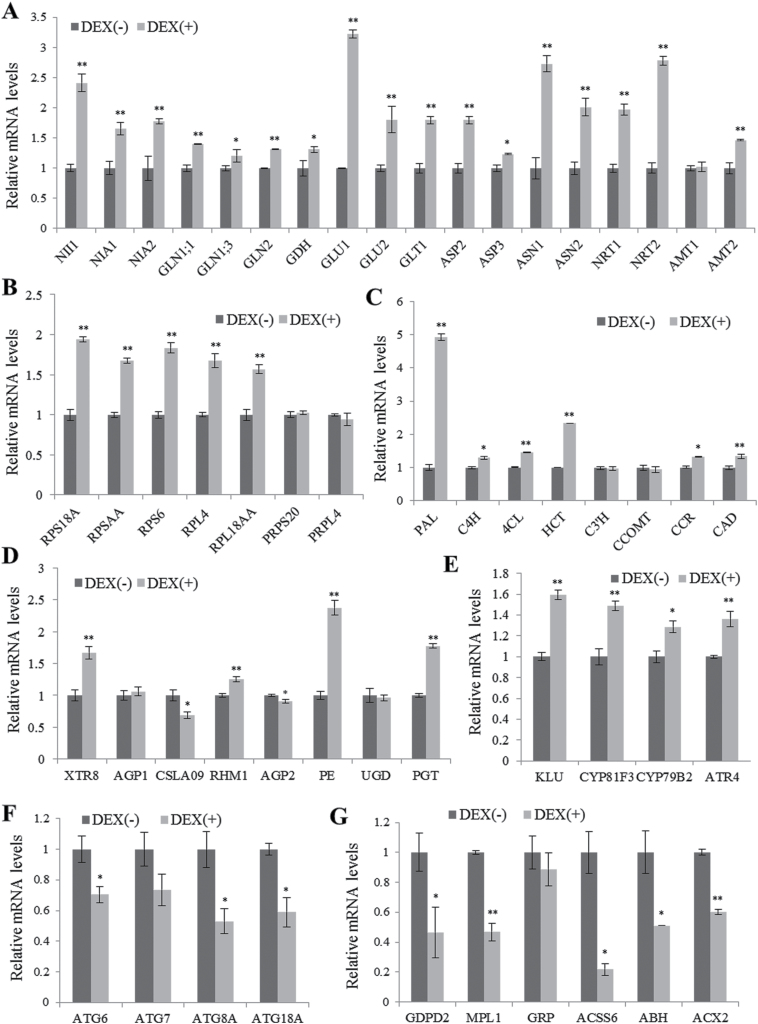
Real-time quantitative RT-PCR analysis of gene expression in DOE-11 seedlings. DOE-11 seedlings grown on MS medium for one week were treated with ethanol [(–)DEX] or 10 µM DEX [(+)DEX] for 3 days for real-time quantitative RT-PCR. Data points represent mean ± SD of three experiments. Asterisks denote statistical significance of the differences between the (–)DEX and (+)DEX samples: *, *P* ≤ 0.05; **, *P* ≤ 0.01. (A) Nitrogen metabolism-related genes. (B) Ribosomal protein genes. (C) Lignin biosynthesis genes. (D) Cell wall biosynthesis genes. (E) Cytochrome 450 genes. (F) Autophagy genes. (G) Lipid degradation-related genes.

To examine changes in gene expression upon induction of *Tap46* overexpression, real-time quantitative RT-PCR was performed using total RNA from DOE-11 seedlings after 3 days of ethanol [(–)DEX] or DEX [(+)DEX] treatment (Fig. 7; Supplementary Table S2). The focus of this analysis was on genes associated with nitrogen metabolism, ribosome biogenesis, lignin biosynthesis, cell wall biogenesis, cytochrome 450, autophagy, and lipid degradation, of which expression was modulated by the TOR pathway (Xiong *et al.*, 2013). Among nitrogen metabolism genes, *NII1* (nitrite reductase), *GLU1* (glutamate synthase), *ASN1* (asparagine synthetase), and *NRT2* (nitrate transporter) genes were highly upregulated, suggesting increased nitrogen assimilation in (+)DEX seedlings (Fig. 7A). A few nuclear-encoded ribosomal protein genes were also upregulated upon DEX treatment, suggesting enhanced ribosome biogenesis processes, while plastid-encoded ribosomal protein genes (*PRPS20* and *PRPL4*) were not affected (Fig. 7B). Among the lignin biosynthetic genes, transcript levels of *PAL* (phenylalanine ammonia-lyase), the first gene of the monolignol biosynthetic pathway, and *HCT* (hydroxycinnamoyl-CoA:shikimate hydroxylcinnamoyl transferase) significantly increased upon DEX treatment (Fig. 7C). Out of eight genes coding for synthesis and modification of cell walls, *PE* showed significantly increased transcript levels upon DEX treatment, while *XTR8* and *PGT* were moderately induced (Fig. 7D). The transcript levels of four cytochrome 450 genes, *KLU*, CYP81F3, CYP79B2, and *ATR4*, were slightly higher in DEX-treated seedlings than in (–)DEX samples (Fig. 7E). *KLU* (CYP78A5) acts in the generation of a mobile growth-promoting signal, while CYP81F3, CYP79B2, and *ATR4* (CYP83B1) are involved in auxin metabolism (Bak *et al.*, 2011). In contrast, autophagy and lipid degradation-related gene transcripts were reduced in DEX-treated seedlings (Fig. 7F, G). These upregulated and downregulated genes in Tap46 overexpression seedlings mimic those induced by TOR activation (Xiong *et al.*, 2013), further supporting the role of Tap46 in the TOR pathway.

## Discussion

Organ growth in plants is controlled by both genetic factors and environmental conditions that coordinate cell division and cell expansion in a spatial and temporal manner (Horiguchi *et al.*, 2006; Anastasiou and Lenhard, 2007; Krizek, 2009). *Arabidopsis* leaf growth consists of proliferation, expansion, and maturation phases. In the proliferation phase, cell division and expansion occur simultaneously. In the expansion phase, cells cease to divide, but continue to expand. In the maturation phase, cells no longer divide or expand (De Veylder *et al.*, 2001; Sonoda *et al.*, 2009; Gonzalez *et al.*, 2010). Genetic determinants of organ growth have been identified, and any perturbation in gene expression of one of these determinants may affect final organ size (Busov *et al.*, 2008). Among the determinants, the plant hormones auxin and brassinosteroid (BR) are important plant growth regulators that stimulate both cell division and expansion. *ARGOS* and *AINTEGUMENTA* encode transcription factors involved in the auxin signalling pathway. Loss-of-function mutations of either gene result in smaller organs, while overexpression results in plant enlargement (Elliott *et al.*, 1996; Mizukami and Fischer, 2000; Hu *et al.*, 2003). ARF2, an auxin response factor, functions as a negative regulator of organ growth: *arf2* mutant plants exhibit larger seeds, embryos, leaves, and flowers (Schruff *et al.*, 2006). ARF2 mediates the crosstalk between auxin and BR signalling pathways that act synergistically in cell elongation (Vert *et al.*, 2008). In addition, overexpression of gibberellin metabolism and response genes, TCP (TB1, CYC, and PCF) domain transcription factors, or growth-regulating factors (GRFs), also result in increased organ size (Busov *et al.*, 2008).

The TOR pathway regulates cell growth in response to nutrient status and stress, thus linking final organ size and environmental conditions. *Arabidopsis* plants that overexpress *TOR* exhibit larger leaves and longer roots (Deprost *et al.*, 2007), although expression of even higher levels of *TOR* results in pleiotropic defects in plant development (Ren *et al.*, 2012). In contrast, RNAi of *TOR* or mutations of LST8/GβL, a partner of TOR, results in retarded seedling growth and decreased organ size (Deprost *et al.*, 2007; Moreau *et al.*, 2012; Xiong and Sheen, 2012). In this study, overexpression of Tap46 increased final organ size, including leaves, siliques, and seeds, without any negative pleiotropic effects under normal growth conditions. Analysis of cell number and cell area revealed that increased leaf size of Tap46 overexpression plants was mainly due to increased cell size, although cell number also increased to a small degree (Fig. 2). These results suggest that Tap46 plays a role in the regulation of both cell expansion and cell proliferation.

Xiong and Sheen (2012) recently reported a sensitive assay for monitoring endogenous TOR activity based on S6K phosphorylation. They found that TOR kinase phosphorylates the Thr-449 and Thr-455 residues of S6K1 and S6K2, respectively, in fully differentiated leaf cells. Additionally, either inactivation of TOR by rapamycin or *TOR* silencing by RNAi abrogated S6K phosphorylation. Considering that S6K is a key substrate and mediator of TOR kinase in yeast and mammals (Wullschleger *et al.*, 2006; Ma and Blenis, 2009), the TOR-S6K1 paradigm appears to be conserved in plants as well. Furthermore, the phytohormone auxin triggers TOR activation leading to S6K1 phosphorylation on Thr-449 and efficient loading of uORF mRNAs onto polysomes, directly connecting the plant TOR/S6K pathway with translation (Schepetilnikov *et al.*, 2013). Here, Tap46 overexpression and *Tap46* RNAi increased and decreased S6K1 phosphorylation on Thr-449, respectively, supporting a role of Tap46 as a positive regulator of the TOR pathway (Fig. 6A, B).

Consistently, Tap46 protein levels decreased following estradiol-inducible *TOR*- RNAi or by rapamycin treatment (Fig. 6F, G). In contrast, PP2Ac protein levels increased when TOR activity was compromised by either method (Fig. 6F, G). Tap46 interacts with and sequesters PP2A catalytic subunits. Thus, Tap46 can modulate the formation of PP2A holoenzyme complexes comprised of the catalytic C subunit, the scaffolding A subunit, and the regulatory B subunit (B, B’, and B’’). Therefore, a decrease in Tap46 protein and an increase in PP2Ac in a cell may result in increased formation of PP2A holoenzyme complexes, subsequently stimulating cellular dephosphorylation activity. In yeast, Tap42 phosphorylation by TOR kinase correlates with Tap42 association with phosphatase catalytic subunits (Jiang and Broach, 1999). TOR inactivation induces dephosphorylation of Tap42 and the release of phosphatases from Tap46, followed by increased dephosphorylation of downstream targets of TOR signalling, supporting the hypothesis that Tap42 functions as a phosphatase inhibitor (Jacinto *et al.*, 2001; Dűvel and Broach, 2004). However, mammalian α4 associated with different phosphatase catalytic subunits exhibits opposing allosteric effects on phosphatase activity depending on the substrate, suggesting that α4 can function as a phosphatase activator (Prickett and Brautigan, 2006; Nien *et al.*, 2007). The PP2A family of phosphatases control diverse cellular processes, including cell cycle control, transcription, translation, and metabolism (Janssens and Goris, 2001; Farkas *et al.*, 2007). This study suggests that Tap46 mainly acts as an inhibitor of PP2A activity in plants, but detailed mechanisms of Tap46 modulation of PP2A activity, both direct and indirect, require further study.

Using BiFC and co-immunoprecipitation, we demonstrated that Tap46 interacts with S6K1 and S6K2 in *Arabidopsis* (Fig. 6C-E). In mammalian cells, S6K is dephosphorylated by PP2A and interacts with PP2A (Ferrari *et al.*, 1993; Peterson *et al.*, 1999; Westphal *et al.*, 1999). Furthermore, S6K1 is associated with the heterodimer of α4 and PP2Ac in mouse spleen B cells and α4 modulates S6K activity through negative regulation of PP2A activity upon B-cell activation (Yamashita *et al.*, 2005). Whether Tap46 negatively regulates PP2Ac on plant S6Ks in a similar manner remains to be tested. Tap46 has multiple phosphatase partners, including catalytic subunits of PP2A, PP4, and PP6, and is a major regulator of cellular PP2A activities in plant cells (Ahn *et al.*, 2011; Hu *et al.*, 2014). Recently, Tap46 was shown to play a role in ABA-regulated seed germination through interaction with the ASCISIC ACID INSENSITIVE5 (ABI5) transcription factor in *Arabidopsis* (Hu *et al.*, 2014). Constitutive overexpression of *Tap46* and weak alleles of the *tap46* mutation resulted in increased and decreased ABA sensitivity during seed germination, respectively. *Tap46*-overexpressing seeds contained increased transcript levels of late embryogenesis-abundant genes, *AtEm1*, *AtEM6*, and *LeaD34*, of which *AtEm1* and *AtEM6* are known to be ABI5-regulated genes (Hu *et al.*, 2014). Furthermore, expression of stress-inducible genes such as *RD29A*, *RD29B*, and *NCED3* was induced at higher levels in *Tap46*-overexpressing plants upon ABA treatment compared with WT plants, suggesting that Tap46 acts as a positive regulator of ABA-regulated gene expression (Hu *et al.*, 2014). We observed in this study that the Tap46 OE-5 and OE-10 seeds are more resistant to environmental stresses such as high temperature and high humidity, based on the survival rate and germination rate after stress treatment (Fig. 3E, F). The enhanced resistance of the Tap46 overexpression seeds may be caused by their enhanced ABA sensitivity for regulation of ABA downstream genes under stress conditions as well as during seed maturation. It is noteworthy that Hu *et al.* (2014) did not observe enhanced growth in their *Tap46* overexpression *Arabidopsis* lines, while immunoblotting with anti-Tap46 antibodies detected ~40% increases in the endogenous level of Tap46 proteins in the plants. We detected a ~4-fold increase in cellular Tap46 protein levels in seedlings of the overexpression lines (Fig. 1G), which may be responsible for the phenotype observed in this study.

Tap46 overexpression caused upregulation of a subset of genes related to nitrogen metabolism, ribosome biogenesis, and lignin biosynthesis, and downregulation of several genes related to autophagy and lipid degradation. This pattern of gene expression correlates with the pattern caused by TOR activation (Xiong *et al.*, 2013), supporting a functional link to the TOR pathway. Transcriptome analysis of transgenic *Arabidopsis* lines overexpressing *AVP1*, *GRF1*, *JAW*, *BRI1*, or *GA20OX1*, all of which exhibit increased leaf size due to different mechanisms, previously showed that all five genes affect the transcriptome distinctly, suggesting that enhanced organ growth is controlled by multiple, possibly independent, pathways (Gonzalez *et al.*, 2010). Nonetheless, kinematic analysis of leaf growth revealed that increased cell proliferation is mainly responsible for leaf-size enlargement in all of the five overexpression lines (Gonzalez *et al.*, 2010). The leaf enlargement in *Tap46* overexpression seedlings is predominantly caused by increased cell expansion (Fig. 2); also the *Tap46* overexpression-induced gene expression profile, although analysed on a limited scale, did not seem to match any of the five lines. These results suggest that the Tap46-mediated growth-regulating pathway may function independently in organ-size regulation.

## Supplementary material

Supplementary data can be found at *JXB* online.

Supplementary Table S1. Data points for the kinematic analysis shown in Fig. 2.

Supplementary Table S2. PCR primers used in this study.

Supplementary Table S3. Measurement of NR activation state.

Supplementary Figure S1. Representative epidermal cells of the first leaves of WT, OE-5, and OE-10 plants at 4 to 25 DAC.

Supplementary Figure S2. Sizes of the epidermal cells at 2 DAC.

Supplementary Figure S3. Quantification of the band intensity in western blots.

Supplementary Figure S4. Control experiments for BiFC.

Supplementary Figure S5. Subcellular localization of S6K2 in *N. benthamiana* leaves.

Supplementary Methods S1. Materials and methods.

## Funding

This research was supported by the Cooperative Research Programme for Agriculture Science & Technology Development [Project numbers PJ009079 (PMBC) and PJ00952503 (SSAC)] from the Rural Development Administration of the Republic of Korea. CSA was supported by the Research Fellow Scholarship (NRF-2013R1A1A2062026) from the National Research Foundation of the Republic of Korea.

## Supplementary Material

Supplementary Data
