# Supplementary material for: Overexpression of the PP2A regulatory subunit Tap46 leads to enhanced plant growth through stimulation of the TOR signalling pathway
Source: J Exp Bot. 2014 Nov 15;66(3):827–40. doi: 10.1093/jxb/eru438 (PMC4321543; doi:10.1093/jxb/eru438)
Supplement: Supplementary Data [file supp_66_3_827__index.html]

Overexpression of the PP2A regulatory subunit Tap46 leads to enhanced plant growth through stimulation of the TOR signalling pathway — Overexpression of the PP2A regulatory subunit Tap46 leads to enhanced plant growth through stimulation of the TOR signalling pathway — Supplementary Data 

# Overexpression of the PP2A regulatory subunit Tap46 leads to enhanced plant growth through stimulation of the TOR signalling pathway

## Supplementary Data

Data files

**Files in this Data Supplement:**

- Supplementary Data - Supplementary Data
