# Supplementary material for: Overexpression of the PP2A regulatory subunit Tap46 leads to enhanced plant growth through stimulation of the TOR signalling pathway
Source: J Exp Bot. 2014 Nov 15;66(3):827–40. doi: 10.1093/jxb/eru438 (PMC4321543; doi:10.1093/jxb/eru438)
Supplement: Supplementary Data [file supp_eru438_jexbot127670_file001.pdf]

Supplementary Figure S1

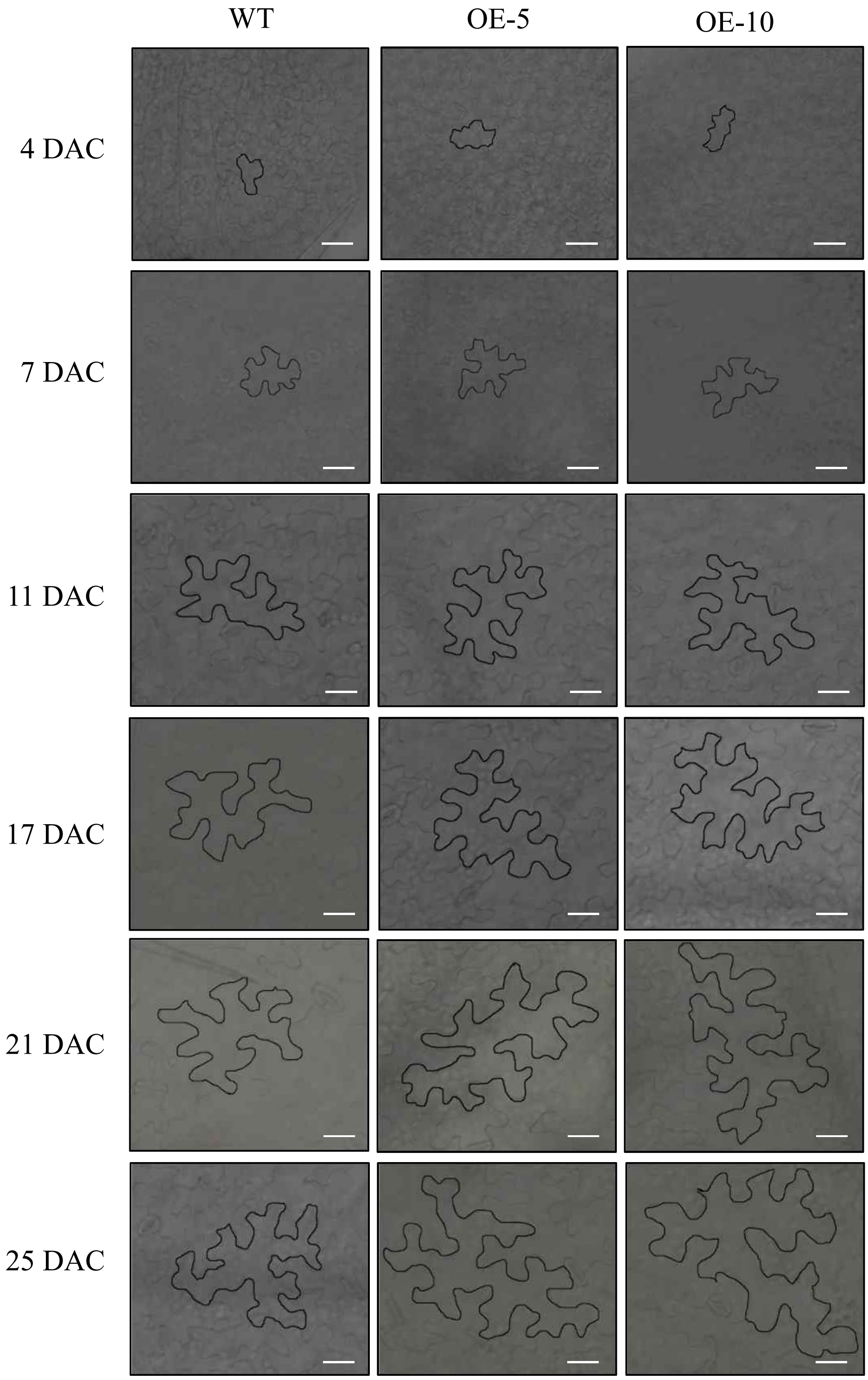

Supplementary Fig. S1. Representative epidermal cells of the first leaves of wild-type, OE-5 and OE-10 plants at 4 to 25 days after cotyledon emergence (DAC). Individual cells were visualized by *black outlines* using ImageJ program. Scale bars = 20 µm.

Supplementary Figure S2

A

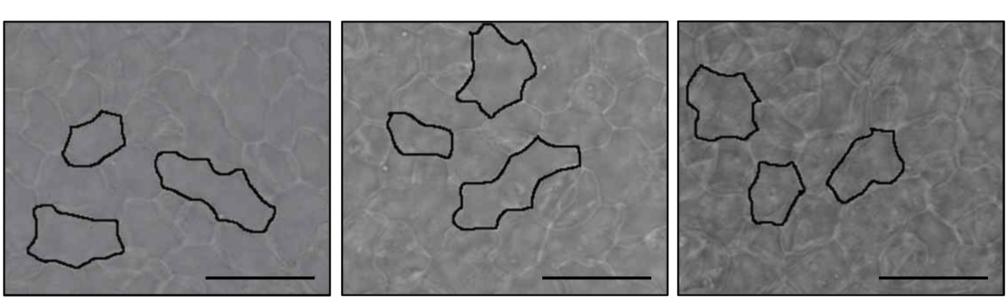

B

	WT	OE-5	OE-10
Average cell area at 2 DAC (μm^2)	166.34±47.05	167.77±44.19	166.41±48.65

Supplementary Fig. S2. Sizes of the epidermal cells at 2 days after cotyledon emergence (DAC).

(A) Representative epidermal cells of the first leaves of wild-type, OE-5 and OE-10 plants at 2 DAC. Scale bars = 20 μm.

(B) Average areas of epidermal cells on the abaxial side of the first leaves at 2 DAC (n = 40).

Supplementary Figure S3

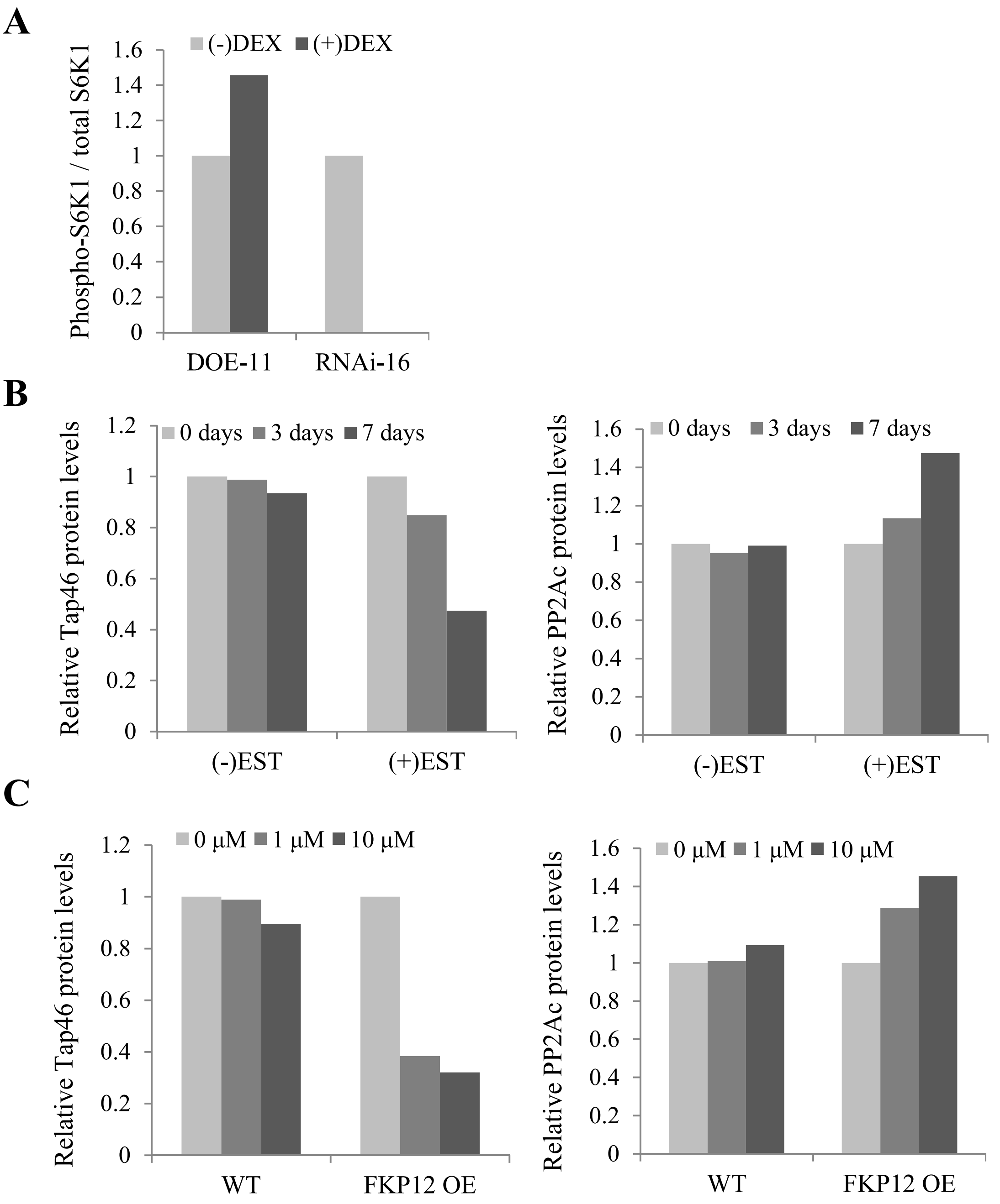

Supplementary Fig. S3. Quantification of the band intensity in Western blots using ImageJ.

(A) Ratio between phosphorylated S6K1 and total S6K1 in DOE-11 and RNAi-16 lines based on the immunoblot band intensities in Fig. 6A and 6B.

(B) Relative Tap46 and PP2Ac protein levels in *TOR* RNAi plants after ethanol (-EST) or 10 μM estradiol treatment (+EST) based on the immunoblot band intensities in Fig. 6F. Rubisco large subunit levels were used as a control for quantification.

(C) Relative Tap46 and PP2Ac protein levels in rapamycin-treated WT and *FKP12* OE lines based on the immunoblot band intensities in Fig. 6G.

Supplementary Figure S4

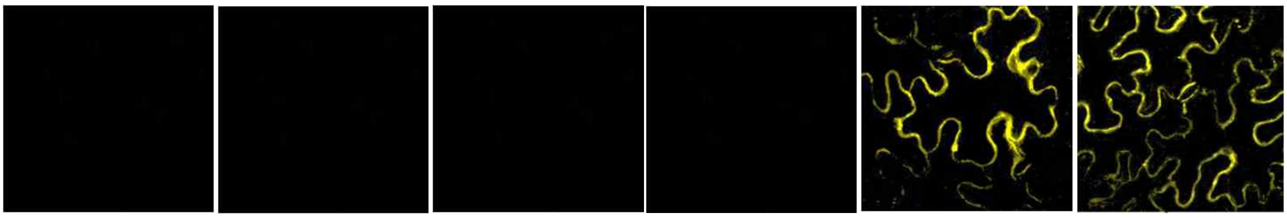

Supplementary Fig. S4. Control experiments for BiFC.

For BiFC, YFP^{N} or YFP^{N}-fusion proteins were expressed together with YFP^{C} or YFP^{C}-fusion proteins in *N. benthamiana* leaves by agroinfiltration. Then epidermal cells of the infiltrated leaves were observed by confocal laser scanning microscopy to detect YFP fluorescence. No YFP fluorescence suggests lack of protein interaction.

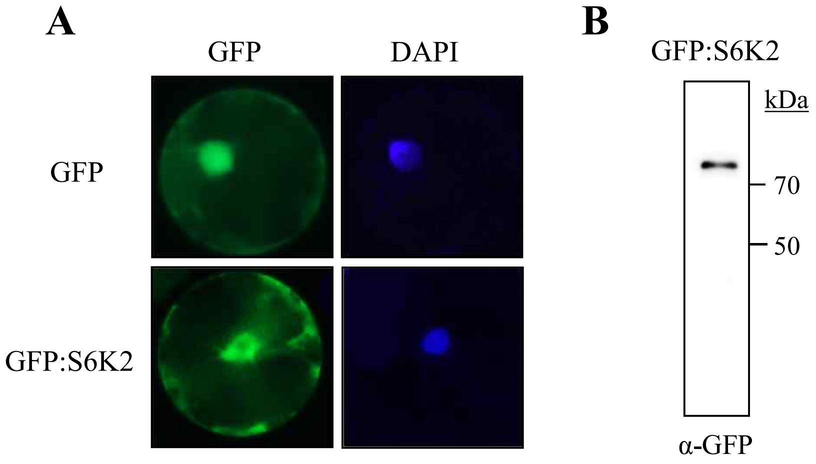

Supplementary Fig. S5. Subcellular localization of S6K2 in *N. benthamiana* leaves.

(A) GFP and GFP:S6K2 proteins were expressed in *N. benthamiana* leaves using agroinfiltration. Protoplasts prepared from the infiltrated leaves were observed by fluorescence microscopy after brief staining with DAPI to visualize nuclei.

(B) Expression of GFP:S6K2 proteins in the infiltrated leaves was determined by Western blotting with anti-GFP antibody.

Supplementary Table S1. Data points for the kinematic analysis shown in Fig. 2.

A

	Average areas of first leaves (mm^2)		
	WT	OE-5	OE-10
4 DAC	0.109±0.016	0.209±0.023	0.248±0.019
7 DAC	0.960±0.0615	1.863±0.122	1.979±0.13
11 DAC	4.582±0.557	7.591±0.591	9.045±0.881
17 DAC	8.06±0.851	14.013±0.989	15.651±0.946
21 DAC	8.183±0.973	17.114±0.819	19.572±1.348
25 DAC	8.641±0.67	17.315±0.614	20.030±0.956

B

	Average areas of epidermal cells (μm^2)		
	WT	OE-5	OE-10
4 DAC	223.009±64.4	301.575±99.1	329.114±144.7
7 DAC	647.199±166.7	814.802±291.8	827.181±210.2
11 DAC	1147.211±310.4	1611.467±535.9	1962.362±598.3
17 DAC	1970.779±651.7	2728.974±893.9	2938.553±776.6
21 DAC	1954.657±831.6	3364.956±941.2	3500.792±824.4
25 DAC	2069.310±744.1	3345.654±631.1	3627.18±892.2

C

	Calculated numbers of epidermal cells per leaf		
	WT	OE-5	OE-10
4 DAC	492.506	693.8004	756.4243
7 DAC	1484.033	2287.465	2393.389
11 DAC	3994.615	4710.821	4609.58
17 DAC	4089.772	5135.142	5326.204
21 DAC	4186.558	5086.162	5590.734
25 DAC	4175.925	5175.435	5522.314

Supplementary Table S2

PCR primers used in this study

Primer name	Gene ID	Description	Sequence (F)	Sequence (R)
UBC10	AT5G53300	ubiquitin-conjugating enzyme 10	Atgggtccttcagagagtcct	tggaacaccttggtcctaaag
Tap46	AT5G53000	PP2A regulatory subunit	ccagatgatgacagcgaaga	tccaacagatcaatagccttacaa
TOR	AT1G50030	target of rapamycin	tcacgacattggatttggaat	aactgctagctccaagtcacg
NII1	AT2G15620	nitrite reductase	tcacaaggaaaccccga	agatcatgagtccccaccac
NIA1	AT1G77760	nitrate reductase 1	aagccgtacacattaaaaggcta	tcacctcaaccctcgttacc
NIA2	AT1G37130	nitrate reductase 2	ctttggtagacgccgaactc	tttagctcgttg attatgtactctg
GLN1;1	AT5G37600	cytosolic glutamine synthetase1;1	ccactgacaaaatcattgctg	tcactggtccaggtagagtcct
GLN1;3	AT3G17820	cytosolic glutamine synthetase1;3	ggccctcagggaccttacta	tccacaatgtcacgaccaat
GLN2	AT5G35630	chloroplast glutamine synthetase	gtggaggcaataacatcttgg	tgtttgttggaattggctca
GDH	AT5G18170	glutamate dehydrogenase	gacgcggatgagatcctaag	tcctcccacataaagccttg
GLU1	AT5G04140	glutamate synthase 1	gggaatctcattgctttcaagt	tgacacacttccagtgaatgc
GLU2	AT2G41220	glutamate synthase 2	tccgtggtagtgcatctcaa	aacgtctccctagccagctc
GLT1	AT5G53460	NADH-dependent glutamate synthase	tgttggtctgcctcatgatt	ccagcagaaggaagtacaaagc
ASP2	AT5G19550	cytosolic aspartate aminotransferase 2	ggtgcctaccgaactgagg	gtattccttgacccgagacg
ASP3	AT5G11520	chloroplast aspartate aminotransferase 3	taccgaactgaggagggaaa	accaatccaacaatgggaag
ASN1	AT3G47340	glutamine-dependent asparagine synthase 1	tcgtgttcttgagctttctcg	ccgttctgatataagccactcc
ASN2	AT5G65010	asparagine synthetase 2	ctgtgaagtgattgcacatcttta	gcgaacattccatccaacat
NRT1	AT1G12110	nitrate transporter 1	aatttcctcggaacttctttca	cggcgaatatagcaatcgtt
NRT2	AT1G08090	nitrate transporter 2	tggaaatcgagctaccttgg	gtaacggcataccacagaatctt
AMT1	AT4G13510	ammonium transporter 1	atcatctgcgggttcgtg	gagcttctcagcgagcttgt
AMT2	AT2G38290	ammonium transporter 2	ttccggcggttatgttattc	cttaggccttggtcctaccc
RPS18A	AT1G22780	40S ribosomal protein S18	tgagcgtctcaagaagatcaga	cgcttcttggaaacaccaac
RPSAA	AT1G72370	40S ribosomal protein SA	caagagacgcaacgatggta	gggttctcaatggcaacaat
RPS6	AT4G31700	ribosomal protein S6	gccgcaagttcacaaacaa	tctgaagagtcaatggggtca
RPL4	AT1G35200	60S ribosomal protein L4	tttgttaacttgtgtggcgttt	accatgacttccctgctgat
RPL18AA	AT1G29970	60S ribosomal protein L18A	tgcttaccaaaatctccaagg	cacctcaaccactgtataatcagg
PRPS20	AT3G15190	chloroplast 30S ribosomal protein S20	atgcgtcgccttctcaac	gcagcctcacacacaatcaa
PRPL4	AT1G07320	plastid ribosomal protein L4	cgaggtacggtgttgatgc	caattggaactacgggtgct
PAL	AT2G37040	phenylalanine ammonia-lyase	cgcacttcagaaggaacttattaga	atcggataccggaaaatcct
C4H	AT2G30490	cinnamate-4-hydroxylase	atttcattcctatccttagaccattc	gcttagaactcgcaatttgctt
4CL	AT1G65060	4-coumarate:CoA ligase 3	gtctccctcaagccatcctt	aaggctcattgacaacactgg
HCT	AT5G48930	hydroxycinnamoyl-coenzyme a shikimate/ quinate hydroxycinnamoyltransferase	ggttggggtcgtcctatctt	caaaagacaaaccctcgtatgg
C3’H	AT2G40890	coumarate 3-hydroxylase	tgacatgaagggtcatgatttt	ggacaaacccgtcttccag
CCOMT	AT4G34050	caffeoyl-CoA O-methyltransferase 1	tcttgctctccctgaagacg	cggtaaacccaattcgtaattct
CCR	AT5G14700	cinnamoyl-CoA reductase	ccacacttctgcgtttgttg	ttgcttctaactccgccatt
CAD	AT4G39330	cinnamyl alcohol dehydrogenase	accgtgtcgcaagtttcag	tcttgtccatcatccgttgac
XTR8	AT3G44990	xyloglucan endotransglycosylase/hydrolase 8	tgtcactctttggctcgaca	ccggtatggacgaagagact
AGP1	AT5G55730	arabinogalactan protein 1	gatgcaaagtgtttgctgaga	tgcctccttctaaactctcctg
CSLA09	AT5G03760	nucleotide-diphospho-sugar transferases	agcggtttacattccttcagtc	aaagaccataagatgcaatgacc
RHM1	AT1G78570	rhamnose biosynthesis 1	gaaggttcaggaattggcttc	ctttagcagctcctcgacca
AGP2	AT2G22470	arabinogalactan protein 2	ggttgcttctcctcctcaga	tggagttaatccagcggaag
PE	AT1G57590	pectinacetylesterase family protein	cctatggttggactcactctga	aagacttccatccaaacacaca
UGD	AT5G15490	UDP-glucose 6-dehydrogenase family protein	ccaatgggtccctgaagag	ttggaaagctcggcagac
PGT	AT4G24780	pectin lyase-like superfamily protein	tgaccctgaatctgtcgttg	ccggtcgtacatgagaggtaa
KLU	AT1G13710	cytochrome P450 CYP78A5 monooxygenase	aaggatgaaaagttgtctgattctg	caactgtatctgtccctctaaatatca

Supplementary Table S2 (cont.)

Primer name	Gene ID	Description	Sequence (F)	Sequence (R)
CYP81F3	AT4G37400	cytochrome P450, family 81	ctgcaacaaaatgaaccagagt	cacagcggaagtgtctgttc
CYP79B2	AT4G39950	cytochrome P450, family 79	tgacggatcccaacaaaaag	atgatcggccatcctgtg
ATR4	AT4G31500	autophagy protein 4	accgtgtcgcaagtttcag	tcttgtccatcatccgttgac
ATG6	AT3G61710	autophagy protein 6	gaagactccctgccataaaagtt	gccggaaatagttacacatcg
ATG7	AT5G45900	autophagy protein 7	gtaccgcttgctctgaaacc	gtcttcccagtcgaggttga
ATG8A	AT4G21980	autophagy protein 8A	caatttgtatacgtggttcgt	agcaacggtaagagatccaa
ATG18A	AT3G62770	autophagy protein 18A	ttggccatcaaaagaacacc	cggatcaaactggcatctg
GDPD2	AT5G41080	PLC-like phosphodiesterases superfamily protein	gcagccattagcaagatcaa	actgcctctccgacattgtt
MPL1	AT5G14180	*Myzus persicae*-induced lipase 1	atatcgccgcaaaaacctt	tcccctactaatccacttttcg
GRP	AT5G56100	glycine-rich protein /oleosin	tgtggccgagagattggtat	ctctcaccaccgactcaacc
ACSS6	AT5G16340	AMP-dependent synthetase and ligase family protein	aacatgttatccgcgactca	ggttaagatgttgacgggatg
ABH	AT5G18630	alpha/beta-Hydrolases superfamily protein	catgaccgtgatatggttcct	ctgaaatctttcacccacacc
ACX2	AT5G65110	acyl-CoA oxidase 2	agacaacactgtacttctgcaaca	ccgccttggaacttttcttt

Supplementary Table S3. Measurement of nitrate reductase (NR) activation state

	Samples	$-Mg^{2+}$ assay (pmol/min·µg protein)	$+Mg^{2+}$ assay (pmol/min·µg protein)	NR activation state (%)
WT	(-)DEX-5 days	0.627±0.003	0.568±0.022	90.6
	(+)DEX-3 days	0.632±0.011	0.576±0.013	91.1
	(+)DEX-5 days	0.626±0.012	0.577±0.02	92.2
DOE-11	(-)DEX-5 days	0.626±0.009	0.57±0.01	91.1
	(+)DEX-3 days	0.779±0.023	0.723±0.014	92.8
	(+)DEX-5 days	0.847±0.014	0.8±0.007	94.5

NR activities were measured using protein fractions prepared from WT and DOE-11 seedlings after 3 and 5 days of ethanol [(-)DEX] or 10 µM DEX [(+)DEX] treatment in the presence of Mg^{2+} or EDTA. NR activation state is defined as NR activity measured in the presence of Mg^{2+} as percentage of NR activity measured in the presence of EDTA.

Supplementary Methods S1

Materials and methods

Generation of dexamethasone (DEX)-inducible Tap46 overexpression lines in Arabidopsis

The full-length *Tap46* coding region was PCR amplified using primers (5'-ggatccatgggtggtttggctatg-3' and 5'-ggatccgccacaaggtgtgagttt-3') and cloned into the binary vector pTA7002 (Aoyama and Chua, 1997). The recombinant plasmid containing the *Tap46* overexpression (OE) construct was introduced into *Agrobacterium tumefaciens* (C58C1), and transformed into *Arabidopsis* using *Agrobacterium*-mediated transformation. Transgenic plants were selected on growth media containing hygromycin (30 mg/L). For induction of *Tap46* OE, dexamethasone (Sigma) was added to the medium to a final concentration of 10 μM in ethanol (0.033%) and tween 20 (0.01 % w/v) from 30 mM stock solution.

Agrobacterium-mediated transient expression

Agroinfiltration was carried out as described previously (Lee *et al.*, 2013). Agrobacterial cultures (GV3101) containing various constructs fused to the CaMV35S promoter were adjusted to OD_{600}=0.6 in MES buffer (10 mM MES, pH 7.5, 10 mM $MgSO_4$). The suspension was incubated with acetosyringone for 2–3 h at a final concentration of 150 μM, and infiltrated into leaves of WT *N. benthamiana* plants. In all experiments, *Agrobacterium* C58C1 carrying the 35S:p19 construct was co-infiltrated to achieve maximum levels of protein expression. Expressed proteins were analyzed at 48 h post-infiltration.

Real-time quantitative RT-PCR

Real-time quantitative PCR was carried out as described previously (Lee *et al.*, 2013). List of primers used in this analysis is shown in Supplemental Table S2.

Bimolecular Fluorescence Complementation (BiFC)

The *Tap46* coding region was PCR-amplified and cloned into the pSPYNE vector containing the N-terminal region of YFP (amino acid residues 1-155), resulting in pSPYNE-Tap46. Similarly, S6K1 and S6K2 cDNAs were cloned into pSPYCE vector containing the C-terminal region of YFP (residues 156–239), resulting in pSPYCE-S6K1 and pSPYCE-S6K2. The pSPYNE and pSPYCE fusion constructs were agroinfiltrated together into the leaves of

3-week-old *N. benthamiana* plants as described (Ahn *et al.*, 2011). After 48 h, protoplasts were generated and YFP signal was detected using a confocal microsope (Zeiss LSM510).

Co-immunopreciptation

HA-tagged Tap46 constructs and various Myc-tagged S6K1 constructs were co-expressed in *N. benthamiana* leaves by agroinfiltration. After 48-h incubation, leaf extracts were prepared and co-immunoprecipitation was performed according to Ahn *et al.* (2011). After SDS-PAGE, western blotting was carried out using the monoclonal anti-HA antibody (1:2,500; ABM) and anti-Myc antibody (1:10,000; ABM).

Measurement of enzyme activities

Nitrate reductase assay was performed as described (Ahn *et al.*, 2011). For nitrite reductase assay, seedlings were ground to a fine powder in liquid nitrogen, and then were added the extraction buffer (50 mM potassium phosphate buffer (pH 7.5), 1 mM EDTA, 10 mM 2-mercaptoethanol, 100 μM PMSF, and 5 mg PVP), followed by centrifugation at 10,000 g at 4°C for 15 min. Nitrite reductase assay was performed using the supernatant as described (Takahashi *et al.*, 2001). For glutamine synthetase assay, seedlings were ground to a fine powder in liquid nitrogen, and then were added the extraction buffer (50 mM Imidazole-HC1, pH 7.2, 0.5 mM EDTA, 1 mM DTT), followed by centrifugation at 10,000 g at 4°C for 15 min. Glutamine synthetase assay was performed using the supernatant as described (Rhodes *et al.*, 1975).

Accelerated-aging treatment and tetrazolium staining of seeds

For the accelerated-ageing treatment, seeds were incubated for 0-72 h at 43 °C and 100% relative humidity in a closed bottle as described (Seo *et al.*, 2011). Then the seeds were incubated in a 1% (w/v) aqueous solution of 2,3,5-triphenyltetrazolium chloride (Merck) at 30°C in darkness for 2 days according to the procedure described by Oge *et al.* (2008).

Statistical analyses

Two-tailed Student's *t*-tests were performed using the Minitab 16 program (Minitab Inc.; http://www.minitab.com/en-KR/default.aspx) to investigate the statistical differences between the responses of the samples. Significant differences between control and other samples were indicated by one ($P \leq 0.05$) or two ($P \leq 0.01$) asterisks.

Supplementary References

Rhodes D, Rendon GA, Stewart, GR. 1975. The control of glutamine synthetase level in *Lemnaminor* L. *Planta* **125,** 201-211.

Takahashi M, Sasaki Y, Ida S, Morikawa H. 2001. Nitrite reductase gene enrichment improves assimilation of NO_2 in *Arabidopsis*. *Plant Physiology* **126,** 731-741.
